# Effectiveness of a health education intervention on the use of long-lasting insecticidal nets for the prevention of malaria in pregnant women of Pakistan: a quasi-experimental study

**DOI:** 10.1186/s12936-020-03298-2

**Published:** 2020-06-29

**Authors:** Ramesh Kumar, Midhat Farzeen, Assad Hafeez, Baseer Khan Achakzai, Muskan Vankwani, Manohar Lal, Rabia Iqbal, Ratana Somrongthong

**Affiliations:** 1 Health Services Academy, Ministry of National Health Services Regulation & Coordination, Government of Pakistan, Islamabad, Pakistan; 2 Directorate of Malaria, Ministry of National Health Services Regulation & Coordination, Government of Pakistan, Islamabad, Pakistan; 3 Dow international Medical College Karachi, Karachi, Pakistan; 4 Federal Government Polyclinic Postgraduate Institute, Islamabad, Pakistan; 5 College of Public Health Sciences, Chulalongkorn University, Bangkok, Thailand

**Keywords:** Malaria prevention, Antenatal care, Long-lasting insecticide-treated bed nets, Vector control disease, Awareness and use of bed nets

## Abstract

**Background:**

About one quarter of pregnant women in the population of Pakistan are using long-lasting insecticide-treated bed nets (LLINs) for prevention of malaria. Past research reported that adequate information and education would act as mediator to change behaviour among patients for prevention of malaria infection. The effective use of LLINs would contribute to reduction of disease burden caused by malaria. The aim of this study was to determine the effectiveness of health education on the adoption of LLINs among pregnant women living in Tharparkar, a remote district in Sindh Province, Pakistan.

**Methods:**

A quasi-experimental study design with control and intervention groups was conducted with 200 pregnant women (100 in each group). Women in the intervention group were provided with health education sessions on malaria for 12 weeks, while those in the control group obtained routine information from lady health workers (LHWs). Pre- and post-intervention assessment was done of knowledge about malaria and use of LLIN, which was statistically analysed using descriptive statistics and difference in difference (DID) multivariable regression analysis to test effectiveness of the intervention.

**Results:**

Baseline was conducted with 200 pregnant women. Demographic characteristics were similar in both groups with slight differences in age, education, income, type of latrine, and source of drinking water. There were no significant differences between mean knowledge and use of LLINs scores between groups at baseline. However, the estimated DID value after the intervention was 4.170 (p < 0.01) and represents an increase in scores of knowledge in the intervention group compared to control. Similarly DID value of 3.360 (p < 0.05) showed an increase in use of LLINs score after the intervention which was significant, showing that the intervention had a positive effect.

**Conclusions:**

Results proved that health education could be an effective intervention for improving knowledge and usage of LLINs among pregnant women for the prevention of malaria. Such educational interventions have a positive potential to be implemented at larger scale by incorporating them into routine health sessions provided by health workers.

## Background

Malaria continues to be the most prevalent parasitic infection responsible for the high burden of disease and deaths in low-income countries. About half of the world’s population lives in malaria-endemic areas and pregnant women are considered the high-risk group for malaria transmission [1]. As a result, nearly 435,000 deaths annually and 219 million malaria cases were reported in 2017 globally. Cases were more concentrated in Africa (92%), Southeast Asia (5%) and Eastern Mediterranean Region (2%) [2, 3]. Pakistan is reported 5 million malaria cases and 50,000 malaria-attributable deaths annually [4].

Global health experts have found malaria to be the biggest challenge during pregnancy as it poses greatest threat to mothers and their newborn in low-income countries. In endemic areas, fewer than half of pregnant women are expected to be asymptomatic carriers of parasitaemia through the placenta [5]. Malaria during pregnancy is correlated with multiple health issues including low haemoglobin level, termination of pregnancy, miscarriage, under nutrition, and premature delivery [6–10]. Severity of malarial infection can increase three-fold during pregnancy compared to non-pregnant women, which can lead to mortality in about half of affected pregnant women [11]. In malaria-endemic regions, it is estimated that 25 million women become pregnant per year, among which some 10,000 deaths resulted from the vector-borne disease in sub-Saharan Africa [12]. Moreover, 3.7 millions pregnant women are on high risk of *Plasmodium falciparum* malaria in Pakistan [13]. The majority of infections in Pakistan caused by *Plasmodium vivax*, although infections with *P. falciparum* are increasing and account for about 35–40% of cases [14].

Pakistan is one of the countries where malaria is highly endemic with one million reported cases occur every year. About 98% of the population in Pakistan is exposed to malaria; one-third live in extremely high-risk areas [15]; around 6.5 million suspected cases were screened; the majority of the cases were mainly caused by *P. vivax* (84%), *P. falciparum* (15%), and mixed cases (1%). Annual parasite incidence (API) in Pakistan was 1.7, annual blood examination rate (ABER) 3.0 and total positivity rate (TPR) 5.7, with Sindh Province having the highest number of reported cases. Migration within the country and across international borders, variable transmission, low immune status of the population, climatic changes, poor socio-economic conditions, fragile health system, poor resources, illiteracy, and low use of long-lasting insecticide-treated bed nets (LLINs) are some of the complex contributing factors to the transmission of malaria in Pakistan [16].

Pakistan is one of the four regions in Eastern Mediterranean Region (EMR) where malaria is highly endemic. Health planners regard pregnant women their top priority as this vulnerable group contributes to high mortality rates in Pakistan [15]. It has been proven through previous studies that using LLINs can prevent a significant number of deaths due to malaria [17], and the frequency of malaria cases could be reduced by half among pregnant women by using LLINs [18]. Mosquito-net usage has a positive impact in reducing the reproduction number R, if 75% of the population were to use mosquito nets, malaria could be eliminated in the population [19]. Areas with a high burden of disease can prevent the possibility of transmission between host and vector by using mosquito nets [20]. Lady health workers (LHWs) are trained community-workers who play a vital role in providing maternal neonatal child health (MNCH) alongside malaria and tuberculosis control, polio vaccination related outreach services. LHWs are providing awareness and health education sessions for pregnant women at their home [21]. LHWs in Pakistan have highly contributed in reduction of infant mortality rate through effective health education and awareness of mothers in the community [22]. Hence, this study was conducted to promote and ensure usage of LLINs among pregnant women by LHWs delivering health education regarding prevention of malaria in Tharparkar district, Sindh Province, Pakistan.

## Methods

### Study design

This study followed a quasi-experimental design with control and intervention groups conducted at two Union Councils (UCs) of Tharparkar, a highly vulnerable district of Sindh Province, Pakistan. Each UC comprises several villages, with estimated population of 30,000 that access health services from Basic Health Units in their communities [23]. The study took place from January to November 2019.

### Health education-based intervention

An intervention group of 100 women in one selected UC received one-and-half-hour health education session per week over 12 weeks, a total of 18 h duration. Researchers developed four modules for each quality health education session, which were based on an information-motivation-behavioural skills (IMB) model. Each session followed a separate module. The four quality health education modules covered topics such malaria transmission, clinical features of malaria, complications caused due to malaria during pregnancy, and strategies to prevent malaria during pregnancy. Each session lasted 30 min, and participants and facilitators were oriented during these sessions to promote the use and maintenance of their LLINs, how to prevent malaria, and how to seek medical advice in case malarial symptoms arose. The four modules were named Understanding malaria in pregnancy, Main preventive measures for malaria in pregnancy, Insecticide-treated nets, and the fourth was an interactive named Commitment for malaria prevention during pregnancy. Real stories and scenarios with experiences of LLIN use, as identified from previous studies, were highlighted, followed by brainstorming among participants and facilitator [24–28].

### Sample size calculation

The sample size was calculated with 80% power and alpha error of 0.50 to determine 30% improvement in use of LLINs among pregnant women after the intervention; the desired number was 94 in each group. With an additional number to cover attrition rate and inclusion of equal representation from all groups of pregnant women, a total of 200 pregnant women were recruited for the study at baseline with 100 participants in each group.

### Sampling technique

**Fig. 1:**
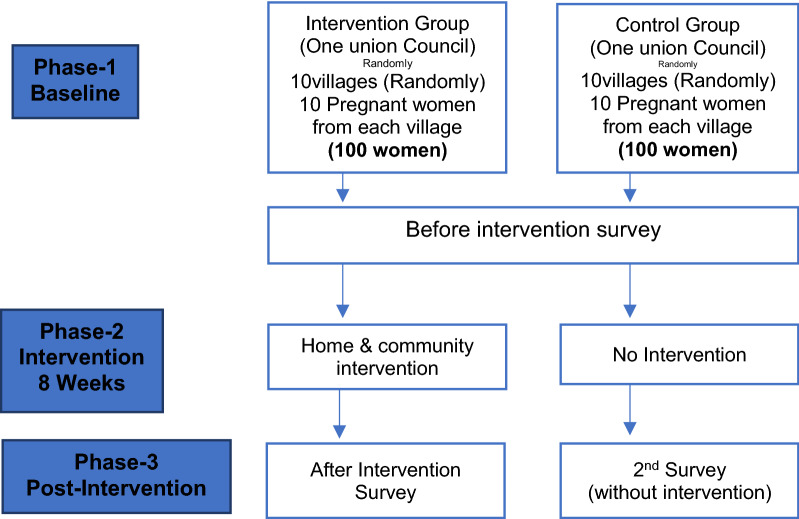
Flow diagram for quasi-experimental study

A multistage, cluster, random sampling technique was used to select study participants. First, the two intervention and control UCs were selected from a list of 44 UCs in the district (primary sampling unit). Next, one UC was assigned to control and the other to intervention. In each UC 10 villages were selected from a list of villages through simple random sampling method (secondary sampling unit) and in each village 10 pregnant women were selected through simple random sampling method from the list provided by the local LHWs. Pregnant women and mothers of children up to 6 months of age were interviewed in their homes. By this method, 200 women were included in the study. Those women who were ill and did not belong to the study area at the time of the interviews were excluded (Fig. 1).

### Data collection

Pre- and post-measurements were made by modifying Malaria Indicator Survey questionnaires developed by Roll Back Malaria Partnership Monitoring and Evaluation Reference Group. The validity and reliability of the tool was established through piloting prior to the start of the data collection process. The tool and health education intervention contents were prepared initially and pre-tested by piloting 20 pregnant women in adjacent UCs with similar kinds of population before the study [29]. The intervention was delivered in the local language and appraised by a midwife, health educationist, experts in this field and an obstetrics and gynaecology specialist for necessary corrections and modifications. Study was conducted with 200 pregnant women (100 in each group). Women in the intervention group were provided with health education sessions on malaria for 12 weeks, while those in the control group obtained routine information from LHWs. The variables included the baseline characteristics and knowledge about malaria of the selected participants at the first contact. Adding scores of total 08 knowledge items generated the knowledge score; the same method was applied for calculating scores of LLIN use by adding 05 variables. Higher scores indicated more knowledge and more use of LLINs and vice versa. Other variable included health-seeking behaviour of participants, training of health care workers, training of pregnant women in management of malaria. Four data collectors were trained before the start of the data collection process. The data collectors introduced themselves to the respondents and explained the objectives of the study. Pattern and time required for interviews was also conveyed to respondents before the start of the interviews.

### Statistical analysis

The statistical analyses were performed to see the effect of several individual characteristics on outcomes of interest (knowledge and use of LLINs). Due to the nature of the experimental design of the study, a difference-in-difference (DID) multivariable regression analysis approach of impact evaluation with both groups, and pre- and post-intervention assessment, was done to check the impact of health education on improved knowledge and the use of LLINs. Baseline survey was conducted before the intervention, and a post-intervention survey was conducted three months after the intervention. Statistical Package Social Science (SPSS version 23) was used for descriptive analysis and STATA was used for DID analysis [30].

### Ethical approval

Ethical approval was obtained from Institutional Ethics Review Board (IERB) at the Health Services Academy, Islamabad, Pakistan (F.No.7/82/2017-IERB). Written informed consent was obtained from all participants in the form of signatures or thumb impressions. The participants were assured that they would not be subject to any undue discomfort during the interview and that they would not receive any monetary incentive for participating in the study. They had been informed of their right to refuse to participate in the study at any time during the interview. The respondents were assured of the confidentiality of the information that they provided.

## Results

At baseline, the age of the women in the control and intervention groups ranged between 18 and 45 years with a mean age of 27. Very few women in control and intervention groups (16 and 13%, respectively) had completed primary school education and most were uneducated. More than 60% of the women were married at or earlier than 18 years of age. More than half of the women in both groups had three to four children. Most women lived in mud houses consisting of three to four rooms (> 0.05), did not own a mobile phone, and did not have an improved source of drinking water, latrine and sewage drainage system in their households. Approximately 35 and 20% women in control and intervention areas, respectively, experienced stillbirths. Similarly, about a quarter of the women said that they had at least one newborn death in the past. Only about a quarter of the women from both groups said that they were counselled by an antenatal care provider about protection against malaria and the commonest topic on which the women in the control and intervention groups received any instruction was indoor residual spraying, 27 and 19%, respectively. Some 32% of women in the control and 21% in the intervention group identified health workers as the most common source of information about malaria. Most women in both groups were aware that malaria was caused by mosquitoes (Table 1).

**Table 1:** Baseline characteristics and information about malaria among control and intervention groups in the study

Variables	Control group (n = 100)	Intervention group (n = 100)	*p* value
Age (years)			
≤ 25	22 (22%)	39 (39%)	0.029*
26–30	53 (53%)	39 (39%)	
31 and above	25 (25%)	22 (22%)	
Education			
Uneducated	62 (62%)	83 (83%)	0.001*
Primary	16 (16%)	13 (13%)	
Any other type of education	22 (22%)	4 (4%)	
Number of living children			
1–2	17 (17%)	10 (10%)	0.111
3–4	54 (54%)	63 (63%)	
5–6	9 (9%)	15 (15%)	
7–9	20 (20%)	12 (12%)	
Age at marriage (years)			
15, 16	12 (12%)	11 (11%)	0.073
17	18 (18%)	34 (34%)	
18	43 (43%)	36 (36%)	
19–22	27 (27%)	19 (19%)	
Type of household			
Mud house	79 (79%)	82 (82%)	0.592
Brick house	21 (21%)	18 (18%)	
Number of rooms			
1–2	30 (30%)	21 (21%)	0.144
3–4	70 (70%)	79 (79%)	
Owns a mobile phone			
Yes	54 (54%)	21 (21%)	0.001*
No	46 (46%)	79 (79%)	
Source of drinking water			
Well outside home	71 (71%)	49 (49%)	0.001*
Well inside home	29 (29%)	51 (51%)	
Type of latrine			
Open	51 (51%)	26 (26%)	0.001*
Pit latrine	49 (49%)	74 (74%)	
Mode of sewage drainage in house			
Open sewers	64 (64%)	33 (33%)	0.001*
Underground sewers	2 (2%)	25 (25%)	
Open pond	34 (34%)	42 (42%)	
Income (PKR)			
5,000	37 (37%)	76 (76%)	0.001*
6000–10,000	48 (48%)	22 (22%)	
11,000–15,000	15 (15%)	2 (2%)	
Previous stillbirth (28 weeks)			
Yes	35 (35%)	20 (20%)	0.018*
No	65 (65%)	80 (80%)	
Previous newborn death			
Yes	26 (26%)	18 (18%)	0.172
No	74 (74%)	82 (82%)	
Antenatal counselling on malaria			
Yes	35 (35%)	24 (24%)	0.088
No	65 (65%)	76 (76%)	
Counselling topics			
Use LLINs	4 (4%)	3 (3%)	0.925
Indoor spray	27 (27%)	19 (19%)	
Take preventive medicine	4 (4%)	2 (2%)	
Malaria during previous pregnancies			
Yes	15 (15%)	29 (29%)	0.017*
No	85 (85%)	71 (71%)	
Malaria during current pregnancy			
Yes	2 (2%)	1 (1%)	0.561
No	98 (98%)	99 (99%)	
Ever heard about malaria			
Yes	34 (34%)	23 (23%)	0.085
No	66 (66%)	77 (77%)	
Source of information about malaria			
Health worker	32 (32%)	21 (21%)	0.683
Other	2 (2%)	2 2(%)	

*Significant (p ≤ 0.05)

Descriptive statistics were calculated for each knowledge and use of LLINs item on both intervention and control groups before the intervention to explore trends regarding use of LLINs between the two groups before and after intervention (Table 2). The women’s perception that malaria was a harmful disease increased from 75 to 97% after the intervention, whereas in the control arm this change was only 10% points from 30 to 40%. The use of LLINs increased in the intervention group at post-intervention assessment due to health education, from 10 to 30%, while in the control group only 1–3% improved. Most of the study population increased their knowledge on mode of transmission from 75 to 97% in the intervention group. Most of the participants heard about mosquito nets after the intervention (58–100%) and their ownership regarding mosquito nets increased up to 76% after intervention (< 0.05). The LHWs were the most prominent source of obtaining malaria information and it significantly increased in intervention group (< 0.05). LLIN use is important in prevention of malaria both in mothers and children and it increased substantially after the intervention (< 0.05). Generally, community knowledge regarding malaria prevention was high. Mosquito net use during pregnancy (practices), importance of LLINs during pregnancy (knowledge) and importance (attitude) increased in both groups; however, a significant change (< 0.05) was observed in intervention group (31–73%). Participants have shown a positive improvement while using indoor mosquito spray after intervention (< 0.05). Most of the respondents reported that they used LLINs the previous night while sleeping; the frequency of LLIN users also increased after intervention (< 0.05). However, no significant differences were found between the groups at baseline except source of information, type of LLINs available and their importance (p > 0.05).

**Table 2:** Comparison of change in the use of long-lasting insecticide-treated bed nets and other malaria preventive measures (before and after, and between control and intervention groups)

Variables	Control (n = 100)			Intervention (n = 100)		
	Before	After	p value	Before	After	p value
Knowledge						
Transmission						
Insect bite	21 (21%)	17 (17%)	0.106	25 (25%)	3 (3%)	0.765
Mosquito bite	79 (79%)	83 (83%)		75 (75%)	97 (97%)	
Symptoms						
Fever	69 (69%)	66 (66%)	0.314	61 (61%)	77 (77%)	0.286
Headache	31 (31%)	34 (34%)		26 (26%)	23 (23%)	
Severity						
Yes	30 (30%)	40 (40%)	0.210	75 (75%)	97 (97%)	0.002*
No	70 (70%)	60 (60%)		25 (25%)	3 (3%)	
Complications						
Fever	69 (69%)	66 (66%)	0.314	61 (61%)	77 (77%)	0.286
Vomiting	31 (31%)	34 (34%)		39 (39%)	23 (23%)	
Prevention						
By using LLINs	46 (46%)	44 (44%)	0.255	31 (31%)	87 (87%)	0.893
Spray	54 (54%)	46 (46%)		69 (69%)	13 (13%)	
Heard about LLINs						
Yes	61 (61%)	48 (48%)	0.065	58 (58%)	100 (100%)	< 0.001*
No	39 (39%)	52 (52%)		42 (42%)	0	
LLINs use						
Protects newborn from malaria	46 (46%)	50 (50%)	0.093	28 (28%)	6 (6%)	0.053*
Protects mother from malaria	54 (54%)	50 (50%)		72 (72%)	94 (94%)	
Use LLINs prevents malaria						
Yes	35 (35%)	40 (40%)	0.465	100 (100%)	100 (100%)	< 0.001*
No	65 (65%)	60 (60%)		0	0	
Use of LLINs						
LLIN present						
Yes	81 (81%)	70 (70%)	0.091	85 (85%)	97 (97%)	0.003*
No	19 (19%)	30 (30%)		15 (15%)	3 (3%)	
Use LLINs in pregnancy						
Yes	35 (35%)	39 (39%)	0.474	31 (31%)	73 (73%)	< 0.001*
No	65 (65%)	61 (61%)		69 (69%)	27 (27%)	
LLINs use important in pregnancy						
Yes	35 (35%)	39 (39%)	0.474	31 (31%)	73 (73%)	< 0.001*
No	65 (65%)	61 (61%)		69 (69%)	27 (27%)	
Use LLINs previous night						
Yes	35 (35%)	36 (36%)	0.883	31 (31%)	96 (96%)	< 0.001*
No	65 (65%)	64 (64%)		69 (69%)	4 (4%)	
Use spray in last 3 months						
Yes	35 (35%)	33 (33%)	0.765	31 (31%)	81 (81%)	< 0.001*
No	65 (65%)	67 (67%)		69 (69%)	19 (19%)	

*Significant (p ≤ 0.05)

DID is usually implemented as an interaction term between time and treatment group dummy variables in a regression model. The coefficient of the treatment variable (intervention), is the estimated mean difference in outcome (knowledge and use of LLINs) between the treatment and control groups prior to the intervention; it represents whatever baseline differences existed between the groups before the intervention was applied to the control group. At baseline, the coefficient of intervention is 0.43 (knowledge) and − 0.02 (use of LLINs), however it is not significant (Table 3).

**Table 3:** Difference in differences (DID) with time trend and interaction term

Variables	Model-1^a^	Model-2^b^	Model-3^c^	Model-4^d^
Treatment	0.430	− 0.020	0.106	− 0.238
	(0.276)	(0.221)	(0.364)	(0.277)
Time (Post-intervention)	0.570**	0.320	0.570**	0.320
	(0.276)	(0.221)	(0.284)	(0.229)
Interaction term	4.170***	3.360***	4.170***	3.360***
	(0.390)	(0.312)	(0.382)	(0.309)
Confounders	No	No	Yes	Yes
R square	0.540	0.500	0.570	0.530
Prob > F	0.000	0.000	0.000	0.000

Confounders: age, education, income, etc

Standard errors in parentheses; *p < 0.1, **p < 0.05, ***p < 0.01

^a^Model 1: Estimation of knowledge without confounders

^b^Model 2: Estimation of use of LLINs without confounders

^c^Model 3: Estimation of knowledge with confounders

^d^Model 4: Estimation of use of LLINs with confounders

The time trend (post intervention) is the expected mean change in outcome from before to after the onset of the intervention among the control group. It reflects the pure effect of the passage of time in the absence of the actual intervention. The coefficient of time trend (post-intervention) is 0.57 (knowledge) and it is significant at 5% level of significance. Similarly, the coefficient of time trend (post-intervention) for use of LLINs is 0.57, which is significant at 5% level of significance (Table 3).

The interaction term by itself is the difference in differences estimator. The coefficient for interaction term is the differences-in-differences estimator. The effect is significant at 1% level of significance with the treatment having a positive effect.

Results of DID regression estimation with an interaction between time (baseline *vs* post-intervention) and groups (control and intervention) were used to the estimate magnitude of the effect of intervention (knowledge and use of LLINs change due to intervention by controlling time) and its statistical significance.

The results (Table 4) showed that there were no significant differences between the mean knowledge and use of LLINs scores of the groups at baseline. However, the mean knowledge and use of LLINs scores increased significantly in the intervention group as compared to the control group after intervention. Therefore, it can be inferred that the estimated DID value of 4.170 (p < 0.01) represents an increase in scores of knowledge in the intervention group compared with control after intervention. Similarly, it is estimated that increase in use of LLINs score after intervention is represented by DID value of 3.360 (p < 0.01). The analysis was also done by controlling for confounders and it showed similar results. Thus, it can be concluded that the scores of knowledge and use of LLINs improved in the intervention group, which is due to the effect of intervention.

**Table 4:** Difference in Differences (DID) Estimation with and without confounders

Difference in differences (DID) without confounders							
Outcome variables	Baseline (BL)			Post-intervention (PI)			Diff-In-Diff
	Control	Intervention	Diff (BL)	Control	Intervention	Diff (EL)	
Knowledge	5.250^a^	5.680	0.430	5.820	10.420	4.600	4.170
SE^b^			0.312			0.235	0.390
t value			1.380			19.57	10.68
p value			0.169			0.000***	0.000***
R square	0.54						
Use of LLINs	4.530	4.510	− 0.020	4.850	8.190	3.340	3.360
SE^b^			0.245			0.194	0.313
t value			− 0.08			17.18	10.75
p value			0.935			0.000***	0.000***
R square	0.59						

Difference in differences (DID) with confounders							
Knowledge	5.605	5.712	0.107	6.175	10.452	4.277	4.170
SE			0.309			0.309	0.383 |
t value			0.35 0			13.82	10.90
p value			0.730			0.000***	0.000***
R square	0.58						
Confounders	Yes						
Use of LLINs	2.785	2.546	− 0.239	3.105	6.226	3.121	3.360
SE			0.250			0.250 |	0.309
t value			− 0.95			12.48	10.87
p value			0.341			0.000***	0.000***
R square	0.53						
Confounders	Yes						

Confounders: age, education, income, etc

***p < 0.01

^a^Means and SE are estimated by linear regression

^b^Robust Std. Errors

## Discussion

Based on baseline information, it was found that participants had some knowledge about malaria but it was inadequate. Their existing knowledge regarding causes and symptoms of malaria could be due to the routine health campaigns and antenatal counselling received from local LHWs. It was known previously that community workers are the main source of information and can play a vital part in prevention of diseases [22]. However, the post-intervention assessment revealed that mean scores of participants in intervention group increased two-fold showing increase in their knowledge whereas the scores in control group remained unchanged. This increasing trend in knowledge in the intervention group was significant and could be attributed to the effectiveness of health education-based intervention. These findings are very similar with those reported in a study conducted in a neighbouring country [31]. Similar results have been reported from other studies conducted in African countries that showed knowledge scores were higher among people who received intervention about malaria [27, 32, 33].

Most of the respondents in this study had LLINs available at home and were well informed on the symptoms of malaria, however usage was minimal in both groups at baseline. Intervention effectiveness was seen in the form of LLIN use among participants of the intervention group, which doubled after intervention. This increasing trend reflects that with adequate knowledge about malaria and the benefits of using LLINs, usage of LLINs increased significantly in the intervention group. Health education intervention has remained effective in imparting behaviour change by improving knowledge about malaria and the benefits of using LLINs [27]. Bangladesh has successfully conducted a mass distribution of LLINs along with health education sessions and found a significant change on the use of LLINs among communities [34]. These findings are comparable with a previous study which has shown that knowledge-based interventions can result in improving knowledge about symptoms of malaria among most people, and thus more people use nets to prevent this disease [6]. Another study shows that the use of LLINs increased up to 30% after education intervention among a rural population [25]. In the study area, malaria is being transmitted throughout the year and most people report being bitten by mosquitoes at night, usually outside their home. Individuals who spend the majority of their time outside are at greater risk of malarial infection. Many reports suggest that regular use of LLINs could prevent this disease [35].

The improvement in knowledge scores and usage of LLINs by pregnant women in the intervention arm could only be associated with the health education provided to them during this study [35]. As each group was taken from separate UCs, it could be assumed that due to distance between the two areas, chances of contamination were minimized. Furthermore, the DID analysis adds value to the inference that difference in scores between groups is valid and that the increase in knowledge and use of LLINs in the intervention group only is due to the effectiveness of intervention received by pregnant women of this group.

True randomization and limited funds available for the execution of this project were the major limitations in this study. Furthermore, endpoint assessment was done when intervention completed its period of 3 months. The increase in knowledge and usage scores could be claimed as immediate impact of intervention. However, long-lasting effects of intervention and true behavioural change can only be ensured if follow-up assessment is done, which was not the case in this study. Therefore, for this intervention to be claimed as having long-lasting behavioural change outcomes, follow-up assessments should be made in future studies that have similar objectives. Lastly, this research might not have benefited all the pregnant women across the country due to the nature and time constraints for the intervention.

## Conclusion

The results of this study revealed that health education is effective and can improve the use of LLINs among pregnant women for malaria prevention in rural areas of Pakistan. It is recommended that health policy makers and programme authorities include health education in routine sessions provided by LHWs to pregnant women. This could not only be beneficial in preventing malaria and reducing its burden of disease but due to the ease of its implementation at scale, it can also improve maternal morbidity and mortality indicators of Pakistan.

## Data Availability

The datasets used and/or analysed during the current study are available from the corresponding author on reasonable request.

## Abbreviations

TDR: Tropical disease research
EMRO: Eastern Mediterranean regional Office
UC: Union council
LLINs: Long-lasting insecticide-treated nets
LHW: Lady health worker
BHU: Basic health unit
SGS: Small grant scheme
WHO: World Health Organization
